# Interpreting Omics Data Analysis with Large Language Models for Disease Target and Drug Discovery

**DOI:** 10.64898/2026.04.30.721768

**Published:** 2026-05-22

**Authors:** ZIXI XU, Weihang Chen, Wuyu Ren, Tianqi Xu, Somadina Amaechin, Raad Khan, Yixin Chen, Michael Province, Philip Payne, Fuhai Li

## Abstract

In biomedical scientific discovery, synthesizing prior knowledge from the literature is an essential component of interpreting numerical omics data analyses for disease target identification and drug discovery. Large language models (LLMs) alone can rapidly retrieve disease mechanisms from biomedical text, but text-only outputs are general and unreliable for target and drug prioritization without cohort-specific quantitative evidence. Herein, we propose a provenance-aware Text-to-Target framework that couples schema-constrained multi-model LLM retrieval with numeric omics data analysis. The key design is a modality-aware fusion step: candidates are partitioned into overlap-supported anchors, retrieval-only hidden hubs, and network-emergent novelty nodes, then propagated into staged hypothesis and strategy generation under topology constraints. We evaluate the model in Alzheimer's disease (AD) and pancreatic ductal adenocarcinoma (PDAC). In PDAC, the workflow produced a balanced 75-gene candidate universe and a 23-strategy portfolio, with significant DepMap support at both target level and strategy level. In AD, stricter candidate controls yielded a compact 34-gene universe and 14 strategies; under an expanded CRISPRbrain registry, both target-level axes were significant , with strong strategy-level enrichment. Across both diseases, final strategies preserved full provenance closure to the candidate pool, enabling end-to-end auditability from retrieval artifacts to validation outputs. These results support a transferable discovery architecture in which omics evidence constrains biological activity, LLM retrieval expands mechanistic search space, and network-aware fusion preserves interpretability. The framework provides a reproducible basis for dual-disease target prioritization and motivates continuous literature-mechanism concordance with agentic evidence-refresh loops.

## Full Text Availability

The license terms selected by the author(s) for this preprint version do not permit archiving in PMC. The full text is available from the preprint server.

